# A case of mycotic aneurysm due to *Burkholderia pseudomallei*

**DOI:** 10.12669/pjms.292.2815

**Published:** 2013-04

**Authors:** C. H. Ding, S. Hussin, M. N. Tzar, M. M. Rahman, S. R. Ramli

**Affiliations:** 1 C. H. Ding, Department of Medical Microbiology & Immunology, Faculty of Medicine, National University of Malaysia, 56000 Cheras, Kuala Lumpur, Malaysia.; 2 S. Hussin, Department of Medical Microbiology & Immunology, Faculty of Medicine, National University of Malaysia, 56000 Cheras, Kuala Lumpur, Malaysia.; 3 M. N. Tzar, Department of Medical Microbiology & Immunology, Faculty of Medicine, National University of Malaysia, 56000 Cheras, Kuala Lumpur, Malaysia.; 4 M. M. Rahman, Department of Medical Microbiology & Immunology, Faculty of Medicine, National University of Malaysia, 56000 Cheras, Kuala Lumpur, Malaysia.; 5 S. R. Ramli, Department of Pathology, Hospital Raja Permaisuri Bainun, 30990 Ipoh, Perak, Malaysia.

**Keywords:** *Burkholderia pseudomallei*, Mycotic aneurysm, Aortic aneurysm, Microgen GN-ID

## Abstract

*Burkholderia pseudomallei* is an free-living gram-negative bacterium causing melioidosis and is endemic in Southeast Asia. A 56-year-old diabetic construction worker with a 1-month history of abdominal pain and 1-day history of high-grade fever was found to have a left non-dissecting infrarenal mycotic aortic aneurysm by abdominal computerized tomography scan. Bacteriological examination of his blood yielded *Burkholderia pseudomallei*. The patient was treated with right axillo-bifemoral bypass with excision of aneurysm and high-dose intravenous ceftazidime for two weeks, followed by oral trimethoprim/sulfamethoxazole and oral doxycycline for a minimum of five months.

## INTRODUCTION

*Burkholderia pseudomallei* is considered as a biological threat for its aerosol dissemination and severe impact on human health. Humans typically become infected through contact with contaminated soil and water. The disease caused by this bacterium is known as melioidosis, characterized by skin, visceral, and genitourinary system abscesses. *Burkholderia pseudomallei *associated with mycotic aneurysm is a rare presentation and is found in 1% - 2% of cases related to high rates of morbidity, mortality, and relapse.^1^ The usual etiological agents in mycotic aneurysms are *Staphylococcus aureus *and non-typhoidal *Salmonella *species.^2^ This is a case of mycotic aneurysm due to *Burkholderia pseudomallei *that has rarely been observed. In this presentation, clinical findings, laboratory investigations and management of mycotic aneurysms are described. 

## CASE PRESENTATION

**Fig.1:**
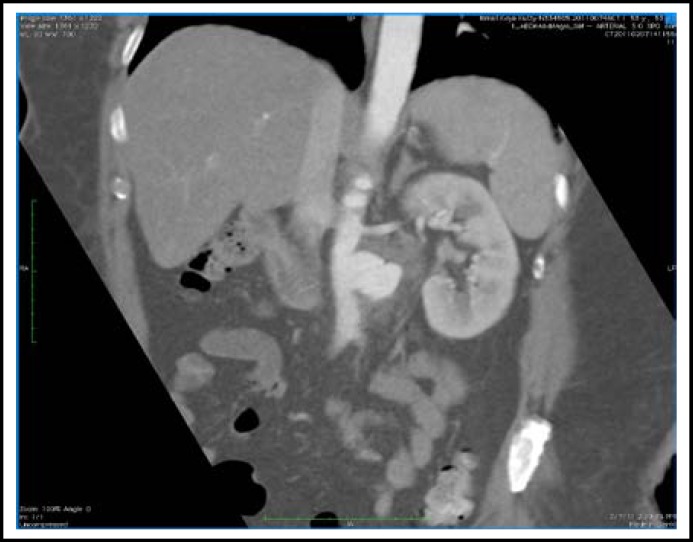
Coronal CT section of the patient’s abdomen showing a left saccular infrarenal aortic aneurysm

**Fig.2:**
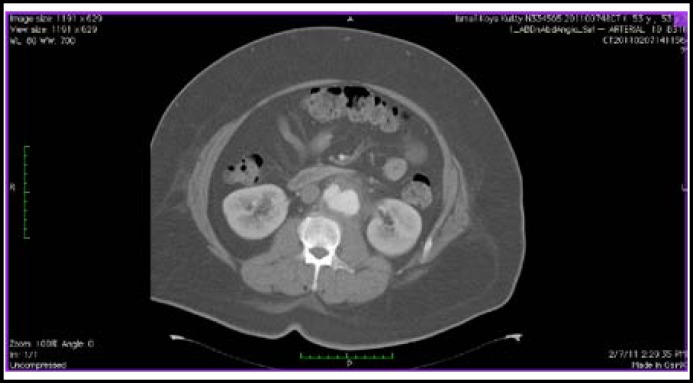
Axial CT section of the patient’s abdomen showing a left saccular infrarenal aortic aneurysm

A 53-year-old diabetic Malay gentleman working as a construction worker had been suffering from left iliac fossa pain radiating to the back for the past one month. There was a marked increase in pain severity four days prior to his initial admission to a private hospital. He was also feverish on the day of admission. A contrasted abdominal CT scan showed a left infrarenal aortic aneurysm. He was then referred to *Universiti Kebangsaan Malaysia Medical Centre* (UKMMC) for further management. On examination, his blood pressure was 130/70 mmHg, pulse rate 96 beats/minute and temperature 38.2^O^C. Respiratory examination was unremarkable. There was tenderness at the umbilical region but no pulsatile abdominal mass was felt. His lower limb peripheral pulses were palpable and equal bilaterally. His total blood leukocyte count was 12.4 x 10^9^ /L, with a predominance of neutrophils. C-reactive protein level of the patient was 30.47 mg/dL. An urgent repeat contrasted abdominal CT examination was done at UKMMC and its findings were similar to that from the private hospital (Fig.1). There was a left saccular infrarenal aortic aneurysm measuring 2.9cm x 3.9cm x 2.6cm at the level of L2/L3 (Fig.2). No dissection or thrombosis was detected. There was also no extravasation of contrast to suggest any active leak. 

**Fig.3:**
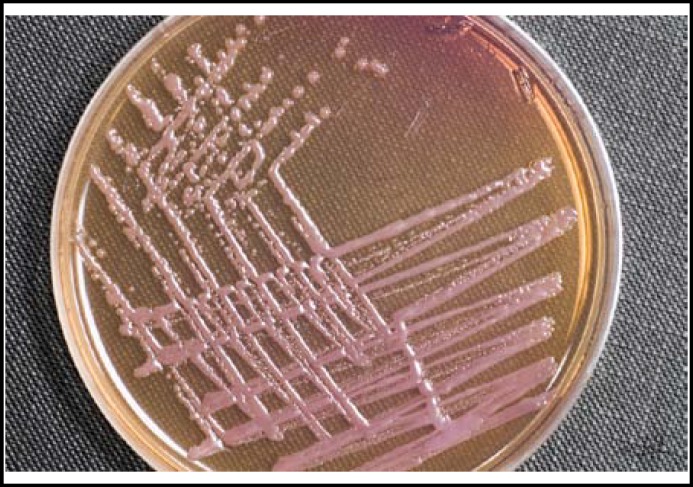
Non-lactose fermenting bacteria colonies on MacConkey agar exhibiting the typical metallic violet sheen of *Burkholderia pseudomallei*

A surgical intervention of right axillo-bifemoral bypass with excision of aneurysm was performed. A blood sample and intraoperative tissue specimens were sent to the microbiology lab for culture, identification and antibiotic sensitivity. A non-lactose fermenting gram-negative rod shaped bacterium was isolated from the specimens (Fig.3). It was identified as *Pseudomonas* sp based on its triple sugar iron (TSI) and oxidase reactions of alkaline/alkaline and positive, respectively. Using Microgen GN-ID (Microgen Bioproducts Ltd, UK), the organism was finally confirmed as *Burkholderia pseudomallei. *In-vitro antibiotic susceptibility testing (E-test) showed that the organism was sensitive to ceftazidime, co-trimoxazole, tetracycline and co-amoxyclav. It was resistant to all aminoglycosides.

After detection of *Burkholderia pseudomallei *bacteraemia, the patient was put on intravenous ceftazidime 2 g tds for 14 days. Upon completion of intravenous ceftazidime, he was allowed home with a course of oral trimethoprim/sulfamethoxazole (320 mg/1600 mg) bd and oral doxycycline 100 mg bd. He was advised to take oral antibiotic therapy for a minimum of five months.

## DISCUSSION

This is a case of an abdominal aortic aneurysm that was diagnosed as mycotic due to a co-presentation of fever. However if his blood and tissue specimens were not sent for bacteriological diagnosis, the aetiological agent (*Burkholderia pseudomallei*) may have never been confirmed as the clinical features of *Burkholderia pseudomallei-*associated mycotic aneurysms are similar to those of conditions caused by other bacteria.^3^ Moreover, cardiovascular complications of *Burkholderia pseudomallei *infection resulting in mycotic aneurysms are very rare.^2^ Rather, the lung is the most commonly affected organ in *Burkholderia pseudomallei *infections, either presenting as a primary lung abscess or pneumonia.^4^ However, the patient in this case report had no radiological evidence of pneumonia.

Melioidosis is endemic in South-East Asia and northern Australia. Although reported cases of *Burkholderia pseudomallei-*associated mycotic aneurysms are few in Malaysia, in Thailand it is the most common causative pathogen implicated in mycotic aneurysms (42.5% of cases).^3^ In Taiwan, the most common causative microorganism was non-typhoid *Salmonella *(57%), followed by *Staphylococcus aureus* (14%) and *Mycobacterium tuberculosis *(11%).^5^

As the patient in this case report works in the construction site, it is likely that he contracted the infection from the soil in his workplace. Humans are usually infected by traumatic inoculation of the organism from the soil or rarely, by inhalation or ingestion. Heavy rains and strong winds may cause increased inhalation of *Burkholderia pseudomallei. *The presence of underlying co-morbid diseases, such as diabetes mellitus in this patient, is considered to be a significant risk factor for melioidosis.^6^

A prolonged course of antibiotic therapy plus surgical intervention is considered to be the most appropriate management of *Burkholderia pseudomallei-*associated mycotic aneurysms.^3^ *Burkholderia pseudomallei *is inherently resistant to aminoglycosides due to an efflux system which prevents the accumulation of the antibiotic in the bacterium.^7^ Current antimicrobial treatment recommendations include administration of intravenous antibiotics (ceftazidime or a carbapenem) for at least 10 days, followed by oral antibiotics for at least 12 weeks.^8^ An oral antibiotic regiment consisting of t*rimethoprim, s*ulfamethoxazole and doxycycline (the combination our patient was discharged home with) was found to be as effective as, and better tolerated than the conventional four-drug regimen (with chloramphenicol as the fourth drug).^8^

The present patient was fortunate to have responded well to combinations of surgical and antimicrobial therapy. However, without appropriate management of similar cases the risks of mortality may be as high as 16%-44%.^3^

## CONCLUSION

*Burkholderia pseudomallei *associated with mycotic aneurysm is a rare presentation The patient with a mycotic aneurysm due to *Burkholderia pseudomallei *with risk factors of diabetes mellitus or whose occupation puts him at risk of contracting the organisms should be given an antibiotic regime for effective treatment.
